# G-Protein Coupled Receptor 83 (GPR83) Signaling Determined by Constitutive and Zinc(II)-Induced Activity

**DOI:** 10.1371/journal.pone.0053347

**Published:** 2013-01-15

**Authors:** Anne Müller, Gunnar Kleinau, Carolin L. Piechowski, Timo D. Müller, Brian Finan, Juliane Pratzka, Annette Grüters, Heiko Krude, Matthias Tschöp, Heike Biebermann

**Affiliations:** 1 Institute of Experimental Pediatric Endocrinology, Charité Universitätsmedizin Berlin, Berlin, Germany; 2 Institute of Diabetes and Obesity, Helmholtz Center Munich, German Research Center for Environmental Health (GmbH), Munich, Germany; 3 Department of Metabolic Diseases, Technical University, Munich, Germany; Medical School of Hannover, United States of America

## Abstract

The G-protein coupled receptor 83 (GPR83) is an orphan G-protein coupled receptor for which the natural ligand(s) and signaling pathway(s) remain to be identified. Previous studies suggest a role of GPR83 in the regulation of thermogenesis and the control of circulating adiponectin. The aim of this study was to gain insights into the molecular underpinnings underlying GPR83 signaling. In particular, we aimed to assess the underlying G-protein activated signaling pathway of GPR83 and how this pathway is affected by mutational activation and zinc(II) challenge. Finally, we assessed the capacity of GPR83 for homodimerization. Our results show for the first time that mouse (m) GPR83 has high basal Gq/11 activity without affecting Gi or Gs signaling. Furthermore, we found that, under physiological conditions, zinc(II) (but not calcium(II) and magnesium(II)) potently activates mGPR83, thus identifying zinc(II) as an endogenous molecule with agonistic capability to activate mGPR83. In line with the observation that zinc(II)-ions activate mGPR83, we identified a cluster of ion-binding sensitive amino acids (e.g. His145, His204, Cys207, Glu217) in an activation sensitive receptor region of mGPR83. The occurrence of a constitutive activating mutant and a zinc(II)-binding residue at the N-terminal part corroborate the importance of this region in mGPR83 signal regulation. Finally, our results indicate that mGPR83 forms homodimers, which extend the current knowledge and molecular facets of GPR83 signaling.

## Introduction

G-protein coupled receptor 83 (GPR83), previously identified as JP05, GPR72 or the glucocorticoid-induced receptor (GIR) is an orphan G-protein coupled receptor (GPCR) belonging to the rhodopsin-like class A GPCRs [1]. GPR83 was originally identified as a stress responsive transcript isolated from the murine thymoma cell line WEHI-7TG after stimulation with glucocorticoids or forskolin [2]-[4]. Furthermore, induction of GPR83 mRNA expression upon dexamethasone [5] or amphetamine [6] treatment suggests a potential role in the regulation of the hypothalamus-pituitary adrenal (HPA) axis. To this extent, GPR83 is most abundantly expressed in the murine brain and the thymus [3]. Former studies showed a selective upregulation of GPR83-expression in regulatory T cells (Treg) suggesting a role in development and function of these cells which are important for maintaining immunological tolerance. However recently it was shown, that GPR83 is dispensable for Treg cell development and activity [7]. Within the mouse brain, GPR83 is highly expressed within forebrain limbic system structures, the striatum and different hypothalamic regions [8]. Of appreciable importance, GPR83 expression patterns are similar between rodents and humans [6], [9], and there is 87% sequence identity amongst the two species [10].

Unlike the well-characterized anatomical location of GPR83 expression, the endogenous ligand(s) and molecular underpinnings of ligand-activated signaling remain to be identified. GPR83 has previously been suggested to play a regulatory role in thermogenesis and also to be involved in the control of circulating adiponectin levels [11]. Other reports suggest GPR83 belongs to the neuropeptide Y (NPY) receptor family, based on the nearly 35% sequence identity GPR83 shares with various members of the NPY receptor family and that the C-terminal fragment of NPY binds to GPR83 at a lower affinity as compared to NPY receptor subtypes [10]. Despite these implicated roles in the neuroendocrine control of energy balance and structural similarities to other neuropeptide receptors, the signaling properties of murine GPR83 (mGPR83) have yet to be characterized.

To identify G-protein signaling cascades initiated by mGPR83 signaling, we first tested the capacity to induce a basal tone of the different G-proteins (Gs, Gi, Gq/11). Subsequently, to confirm the identified pathway, a specific mutation was introduced at a highly conserved amino acid in transmembrane helix 6 (TMH6) [12], [13] to constitutively activate the receptor. In the thyroid stimulating hormone receptor (TSHR) pathogenic mutants at Cys636 (e.g. Cys636Trp) in TMH6 were characterized as constitutive activating mutations (CAMs) [14], [15]. This position is localized in the highly conserved Cys^6.47^-Trp^6.48^-Leu/Ala^6.49^-Pro^6.50^ motif of family A GPCR [16] (the numbers are according to the unified system suggested for family A GPCRs by Ballesteros & Weinstein [17]). Assuming similar micro-switch mechanisms in conserved family A GPCRs [18], [19], this cysteine at mGPR83 position 304 was also mutated to tryptophan (Cys304Trp) and tested for constitutive G-protein activation. In addition, we evaluated other hallmark GPCR-specific signaling features, including the capacity for homodimerization and capability of zinc(II)-activated downstream signaling [20]. It is reported that zinc(II) can behave as an agonist, allosteric modulator or inverse agonist at numerous GPCRs and, thus, can diversely affect signaling [20]-[26]. To specify zinc(II)-action on mGPR83, activation by two further divalent cations, calcium(II) and magnesium(II), was also tested. Several GPCRs like the calcium-sensing receptor [27] are known to bind Ca(II).

Altogether, this study aimed to establish new molecular details of mGPR83 signaling in order to contribute further insights into the physiological and pharmacological characteristics of mGPR83.

## Materials and Methods

### Construction of Wild type and Mutant Receptors

*Gpr83* was amplified from murine hypothalamic cDNA, *TSHR* (thyroid stimulating hormone receptor) from human thyroid cDNA, *MC3R* (melanocortin 3 receptor) and *5HTR1B* (5-hydroxytryptamine 1B receptor) from human genomic DNA and *GHSR* (ghrelin receptor) from human cDNA purchased from UMR cDNA Resource Center, Rolla, MO, USA [28], [29]. Receptor-DNAs were cloned into the pcDps expression vector. Hemagglutinin tags were either cloned at the aminoterminal end (NHA) or subsequent to the signal peptide sequence (SP-HA). FLAG-tags were generated at the carboxyterminal ends (C-FLAG). The pcDps[NHA-rM3R (rat muscarinic receptor 3)] construct was kindly provided by Torsten Schöneberg (Institute of Biochemistry, University of Leipzig) and served as basis for all other cloned rM3R-constructs.

Mutant *Gpr83* were generated by site directed mutagenesis with wild type *Gpr83-* or *SP-HA-Gpr83-*pcDps as template.

The correctness of all PCR-derived products was proven by automatic sequencing. The pGL4.30[luc2P/NFAT-RE/Hygro] reporter construct, co-transfected for IP_3_ determination was purchased from Promega (Madison, WI).

### Cell Culture and Transfection

HEK293 (human embryonic kidney) and COS-7 cells were grown in Earĺs minimum essential medium (Earĺs MEM; Biochrom, Berlin, Germany) and in Dulbeccós modified Eaglés medium (DMEM, Biochrom), respectively, supplemented with 10% fetal bovine serum (PAA Laboratories GmbH, Cölbe, Germany), 100 U/ml penicillin, 100 µg/ml streptomycin (Biochrom, Berlin, Germany) and 2 mM L-glutamine (Invitrogen, Paisley, UK) at 37°C and 5% CO_2_.

For measurement of intracellular IP_3_ via reporter gene assay, HEK293 cells were seeded into 48-well plates (5×10^4^ cells/well), coated with poly-L-lysin (Biochrom). For cAMP accumulation assays, COS-7 cells were seeded into 48-well plates (3.75×10^4^ cells/well). Transfection was performed with 83.3 ng of receptor plasmid-DNA/well and 0.9 µl Metafectene^TM^/well (Biontex, Martinsried, Germany). For IP_3_ measurement equal amounts of a reporter construct containing a response element and the firefly luciferase gene under control of the nuclear factor of activated T-cells (NFAT) (Promega, Mannheim, Germany) was co-transfected. Cell surface expression studies were carried out in COS-7 cells, seeded into 48-well plates (3.75×10^4^ cells/well) using 166.7 ng DNA/well and 1.0 µl Metafectene^TM^/well for transfection. Sandwich ELISA for dimerization studies were carried out in COS-7 cells seeded into 6cm-dishes (7×10^5^ cells). Transfection was performed using 3 µg DNA and 8 µl Metafectene^TM^ per dish.

### Investigation of Different Signaling Pathways

Intracellular cAMP levels for determination of Gs or Gi activation were measured in COS-7 cells by AlphaScreen technology [30]. Cells were transfected 24 h after seeding. One day later, transfection mixture was replaced with medium. The hTSHR stimulated with 100 mU/ml bovine thyroid stimulating hormone (bTSH, Sigma-Aldrich, Taufkirchen, Germany) served as Gs positive control [31], [32]. To investigate Gi activity, stimulation with 50 µM forskolin (Sigma-Aldrich) was performed 48 h after transfection. Cells were incubated for 45′ at 37°C and 5% CO_2_ in serum-free DMEM containing 1 mM 3-isobutyl-1-methylxanthine (IBMX, Sigma-Aldrich), in the absence or presence of forskolin. The h5HTR1B co-stimulated with forskolin and 100 nM serotonin (Sigma-Aldrich) served as Gi positive control [33]. After stopping the reaction by aspiration of medium, cells were lysed at 4°C for 2 h on a shaking platform in 100 µl/well lysis buffer containing 5 nM HEPES, 0.1% BSA, 0.3% Tween20 and 1 mM IBMX. 5 µl of each sample were transferred to a 384-well plate. Acceptor and donor beads were added according to the manufacturers’ protocol (Perkin Elmer Life Science, Zaventem, Belgium).

Intracellular IP_3_ levels were determined in HEK293 cells using a luciferase reporter assay. One day after seeding, receptor plasmids and the reporter construct were co-transfected. Transfection mixture was replaced with medium 18 h to 20 h after transfection. Two days after transfection, cells were incubated in serum-free Earĺs MEM in the absence or presence of increasing concentrations of ZnCl_2_ (Sigma-Aldrich), CaCl_2_ and MgCl_2_ (Merck, Darmstadt, Germany; stock solutions were prepared in HPLC Gradient Grade water (J.T.Baker, Deventer, the Netherlands) [34]) at 37°C and 5% CO_2_. Medium without additives contains 1.8 mM Ca(II) and 0.4 mM Mg(II). After stimulation cells were lysed with 100 µl/well 1× Passive Lysis Buffer (Promega). IP_3_ was determined by luciferase activity according to the manufacturer’s instructions (Promega) (Gq/11 positive control hTSHR stimulated with 100 mU/ml bTSH [31], [32]).

### Cell Surface Expression Studies

To investigate cell surface expression in an ELISA system, HA-tagged receptors were transfected one day after seeding. The tag-less GPR83 served as a negative control. Transfection mixture was replaced with medium 18 h to 20 h after transfection. Three days later, cells were washed, paraformaldehyde-fixed and probed with a biotin-labeled anti-HA antibody (Roche Applied Science, Mannheim, Deutschland). Bound biotin anti-HA-antibody was detected by peroxidase-labeled streptavidin (BioLegend, London, UK) in a substrate/chromogen reaction as described [35].

### Homodimerization Studies

Dimerization was measured using the method of sandwich ELISA. Therefore HA- and FLAG-tagged constructs were co-transfected. Cells were harvested 3 days after transfection and solubilized at 4°C (10 mM Tris/HCl, pH 7.4, 150 mM NaCl, 1 mM EDTA, 1 mM DTT, 1% desoxycholat-Na, 1% NP-40 and 0,2 mM PMSF) over night. Lysates were incubated in anti-FLAG antibody (Sigma-Aldrich)-coated 96-well plates for 2 hours. The HA epitope was detected as described above. Total protein concentration of lysates was measured using a bicinchoninic acid (BCA) based protein assay (Thermo Scientific, Bonn, Germany). Heterodimerization of the NHA-tagged hMC3R and FLAG-tagged hGHSR served as positive [28] and the NHA-tagged hGHSR as negative control.

Statistical analyses were performed using the statistical tools implemented in Graph Pad Prism (GraphPad Software, San Diego, California, USA).

### Molecular Homology Modeling of mGPR83

As a result of advanced experimental methods [36] a large number of GPCR crystal structures, among other rhodopsin variants in the group of dopamine, chemokine, adenosine or acetylcholine receptors, have been published in recent years [37]. These crystal structures represent different receptor conformations [36], [38], served as templates for GPCR homology modeling [39] and are also useful tools to improve pharmacological approaches [40]-[43].

The bovine rhodopsin structure was suggested as a template for mGPR83 modeling by already published algorithms concerning best-template selections [44], [45]. The structural mGPR83 homology model described here is based on an inactive rhodopsin conformation (Protein Data Bank entry code 2I35). Amino acid sequences between mGPR83 and rhodopsin overlap especially in biophysical properties and length at the extracellular loop (ECL) 2 (Electronic supplementary material, Fig. S1). The general modeling procedure was performed as recently described [46].

Gaps of missing residues in the loops of the template structure were completed by the ‘Loop Search’ tool in Sybyl 8.1 (Tripos Inc., St. Louis, Missouri, 63144, USA). Side-chains and loops of homology models were subjected to conjugate gradient minimizations (until converging at a termination gradient of 0.05 kcal/mol*Å) and molecular dynamics simulation (2 ns) by fixing the backbone of the TMHs. Finally, the model was minimized without constraints using the AMBER 7.0 Force field. Structure images were produced using PyMOL software (The PyMOL Molecular Graphics System, Version 1.3 Schrödinger, LLC).

## Results

### Identification of the mGPR83 Signaling Pathway

**Figure 1:**
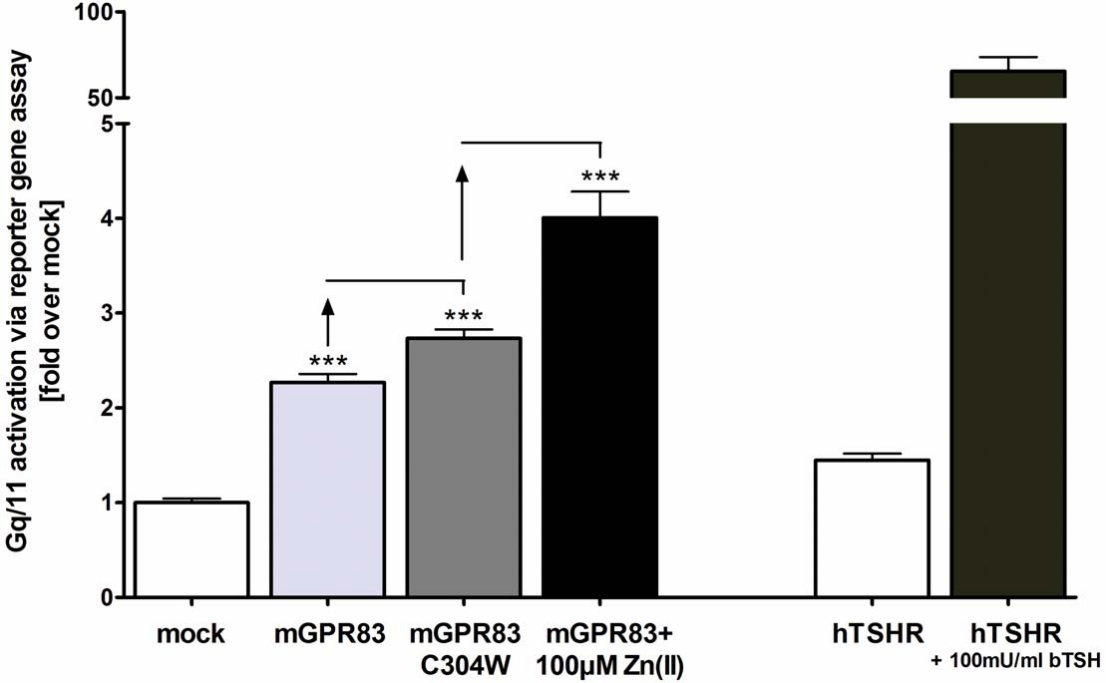
mGPR83 signals via the Gq/11 pathway revealed by basal signaling activity, CAMs and zinc(II)-stimulation. HEK293 cells were transiently transfected with the empty expression vector pcDps (mock) or pcDps carrying the wild type *Gpr83* or the *Gpr83 C304W* mutant, respectively. Two days after transfection, stimulation with ZnCl_2_ (1 nM – 1 mM; stimulation curve in Fig. 2; black column: 100 µM) was carried out and cells were lysed. IP_3_-accumulation was measured in a reporter gene assay. The hTSHR stimulated with 100 mU/ml bTSH functions as assay control [31], [32]. Data were assessed from a minimum of 3 independent experiments, each performed at least in triplicates and represent mean ± SEM calculated fold over basal mock transfection with 19775.4 ± 2259.9 relative light units, set to 1. *** p<0.001 (unpaired t-test, two-tailed).

**Figure 2:**
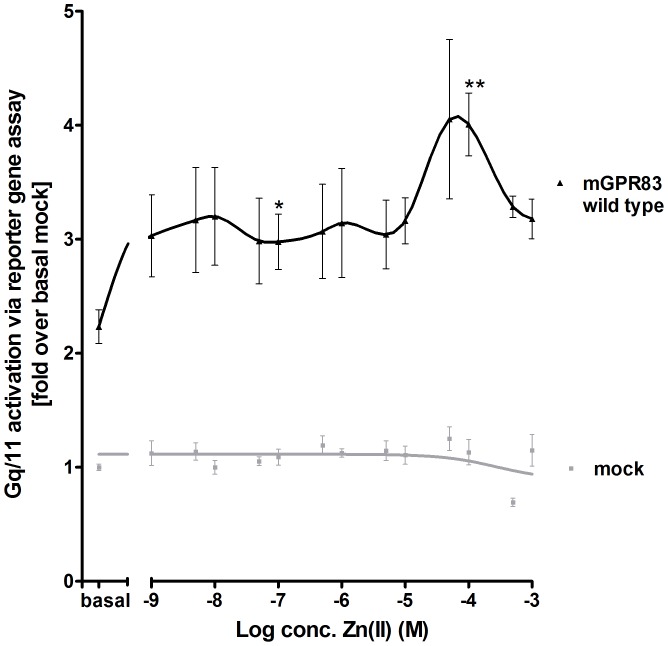
Concentration-response curve of zinc(II)- stimulation at mGPR83. HEK293 cells were transiently transfected with the empty expression vector pcDps (mock) or pcDps encoding the wild type *Gpr83*. Two days after transfection, stimulation with 1 nM – 1 mM ZnCl_2_ was carried out, cells were lysed and IP_3_-accumulation was measured in a reporter gene assay. The hTSHR stimulated with 100 mU/ml bTSH functioned as assay control [data not shown, [31], [32]. The EC_50_ value of the wild type mGPR83 is 10.2 ± 1.4 µM zinc(II) and was obtained from the concentration-response curve (1 nM – 1 mM Zn(II)) using GraphPad Prism. First asterisk indicates a significant increase in IP_3_ formation in comparison to basal wild type. Second asterisks indicate significance in comparison to the first asterisk. Data were evaluated from a minimum of 3 independent experiments, each performed at least in triplicates and calculated fold over the mock transfection, with 24718.3 ± 3958.7 relative light units, set to 1. Shown data represent mean ± SEM. * p<0.05, ** p<0.01 (unpaired t-test, two-tailed).

cAMP accumulation in mGPR83 transiently-transfected HEK293 cells was measured to determine the basal G-protein constituents for mGPR83 signaling. The results revealed that mGPR83 did not alter Gs- or Gi-mediated signal activation compared to the negative empty vector control, the Gs positive control (bovine TSH stimulated hTSHR) or the Gi positive control (forskolin and serotonin co-stimulated h5HTR1B) (Electronic supplementary material, Fig. S2). However, mGPR83 showed a basal Gq/11 signal activity of 2.3 fold compared to mock transfection, as indicated by ligand-independent accumulation of IP_3_ (p<0.001; Tab. 1, Fig. 1 and 2). In support of this notion, the Cys304Trp mutant increased the accumulation of IP_3_ 2.7 fold compared to mock and by 21% compared to baseline levels of wild type mGPR83 (p<0.001; Fig. 1), thus confirming the constitutive activity of this mGPR83 mutant. Furthermore, challenge of wild type mGPR83 with 100 µM zinc(II) further increased IP_3_ formation to a maximum of 4.0 fold compared to mock and by 79% compared to the baseline of mGPR83 wild type (p<0.001; Fig. 1 and 2). This increase in IP_3_ formation (Fig.1 and 2) was consistent with an EC_50_ of 10.2 ± 1.4 µM by treatment with 1 nM – 1 mM zinc(II) (Fig.1 and 2). Interestingly, we observed a biphasic shape of the concentration-response curve (Fig. 2), suggesting binding of zinc(II)-ions at two binding sites and potential allosteric modulation. One first activation step is reached significantly with 100 nM of zinc(II) (p<0.05; Fig. 2). The highest activation level occurs with 100 µM zinc(II) (p<0.01; Fig. 2). Notably, this zinc(II)-mediated activity is strong, with 79% increase over basal mGPR83 signaling compared to zinc(II)-mediated stimulation of other GPCRs (MC4R about 25% referred to mock transfection [23]). Zn(II)-concentrations above 1 mM lead to cell death.

**Table 1:** Functional characterization of amino acids that could be involved in zinc(II)-binding.

Construct	Localization	Cell surface expression	IP_3_ accumulation (Zn(II)-stimulation) fold over basal mock transfection	
		% of wild type	basal	stimulated(1mM Zn(II))
**mock transfection**		8 ± 1	1 ± 0.1	1.2 ± 0.1
**wild type mGPR83**		100 ± 6	2.2 ± 0.1	3.1 ± 0.2
E217A	ECL2	39 ± 2	2.0 ± 0.1	2.0 ± 0.2
D218A	ECL2	74 ± 4	3.2 ± 0.2 ***	3.2 ± 0.2
D227A	ECL2	84 ± 5	2.0 ± 0.1	1.8 ± 0.1
E230A	ECL2	94 ± 7	3.2 ± 0.3 **	2.9 ± 0.3
H27A	Ntt	105 ± 6	3.0 ± 0.1 ***	2.5 ± 0.2
H42A	Ntt	94 ± 6	2.6 ± 0.2	2.6 ± 0.2
H145A	TMH3	74 ± 5	2.0 ± 0.2	2.2 ± 0.2
H204A	TMH4	31 ± 2	1.8 ± 0.1	2.0 ± 0.1
H321A	ECL3	48 ± 4	3.4 ± 0.2 ***	3.3 ± 0.1
H331A	TMH7	68 ± 4	3.2 ± 0.2 **	3.9 ± 0.2
C207A	ECL2	121 ± 7	2.8 ± 0.3	2.5 ± 0.2

For cell surface expression studies COS-7 cells were transiently transfected with the empty expression vector pcDps (mock), *Gpr83* wild type or *Gpr83* mutants. HEK293 cells were used for functional characterization. Data were evaluated from three or four independent experiments, each performed at least in triplicates. IP_3_ accumulation performed as reporter gene assay was calculated fold over the basal mock transfection with 24718.3 ± 3958.7 relative light units, set to 1. The hTSHR stimulated with 100 mU/ml bTSH functioned as control for Gq/11 activation [data not shown, [31], [32]]. Shown data represent mean ± SEM. The mutated aminoacid residues are grouped into extracellular located Ds (Asp) and És (Glu), Hs (His) and one C (Cys) that could be involved in Zn(II)-binding. Asteriks indicate significant higher basal activity in comparison to wild type. ** p<0.01, *** p<0.001 (unpaired t-test, two-tailed); Ntt – N-terminal tail.

Stimulation with Ca(II) more than 1.8 mM and with a Mg(II) concentration more than 0.4 mM did not lead to further mGPR83 signaling activity (supplementary material, Fig. S3).

### Identification of Zinc(II)-binding Sites at mGPR83

**Figure 3:**
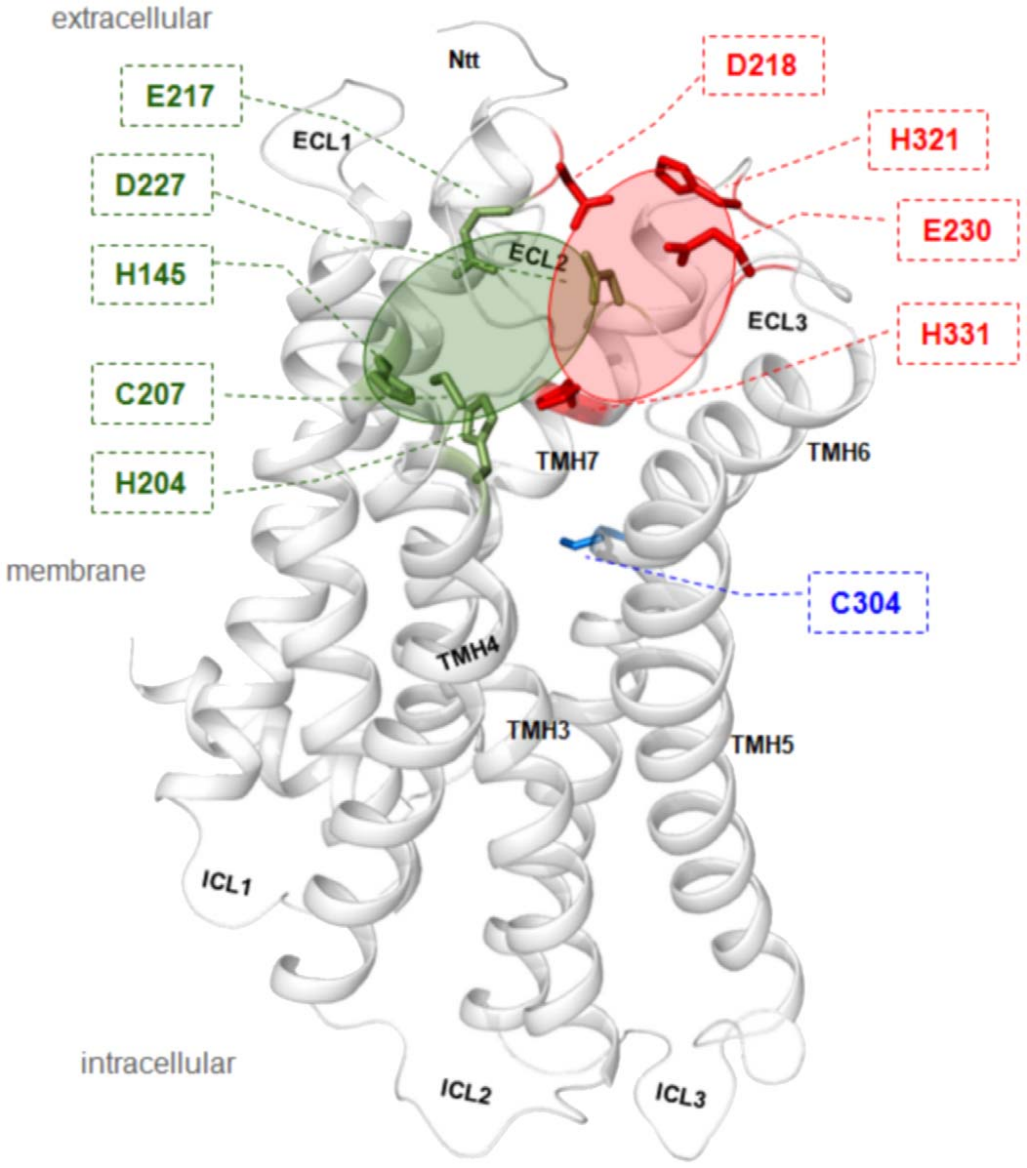
Structural mGPR83 homology model with sensitive positions for constitutive receptor activation and zinc(II)-stimulation. This structual GPR83 homology model is based on the crystal structure of rhodopsin in the inactive state. Highlighted wild type amino acids were depicted from this model for experimental approaches, because they are located extracellularly at the ECLs or at the extracellular ends of the TMHs and those amino acids are known as putative determinants of metal-ion binding motifs: histidine, glutamate, asparagine or cysteine. Arranged in defined spatial arrangements they can interact e.g. with zinc(II)-ions. Interestingly, six side-chain substitutions abolished stimulation by zinc(II) (green sticks) and five substitutions at different positions expressed an increase in constitutive signaling activity of mGPR83 (red sticks). The are spatially clustered in two different regions – red cycle (CAMs) and green (zinc(II)-binding) full cycle. In summary, they are indicating the extracellular region of the mGPR83 as highly sensitive for activation. Cysteine 304 (blue stick) at TMH6 is one of the highly conserved family A GPCR residues and it was reported for several receptors that mutations here are leading almost always to constitutive receptor activation. Indeed, also the mGPR83 Cys304Trp mutation causes a slight ligand independent (constitutive) activation of Gq/11 mediated signaling pathways.

The amino acids potentially involved in zinc(II)-binding at mGPR83 were identified from analyses of the mGPR83 sequence (Electronic supplementary material, Fig. S1) and the structural mGPR83 homology model (Fig. 3). According to previous insights on GPCRs [23], [25], [26], [47], zinc(II) is suggested to bind to extracellular domains including the N-terminal tail (Ntt), the ECLs and the extracellular segments of the transmembrane helices. Amino acids were chosen for mutagenesis (Tab. 1) according to known zinc(II)-ion binding motifs from different protein super-families, which included Cys-Cys-Cys-Cys, Cys-His-Cys, His-His-His or His-Glu-His sequences [48]. To specifically impair zinc(II)-binding capacity, Cys, Glu, Asp and/or His residues in the extracellular region (Fig. 3) were mutated to alanine, and these mGPR83 mutants were functionally characterized for cell surface expression, basal activity and zinc(II)-induced signaling properties. Zinc(II)-stimulation was also performed in concentration-response curves using 1 nM – 1 mM Zn(II). Results of 100 µM and 1 mM Zn(II) were identically in proportion to wild type mGPR83. Therefore and due to the lowest standard error in this saturation region (Fig. 2), only data for 1 mM Zn(II)-stimulation were displayed in table 1. The results revealed that alanine mutations at six residues, His42, His145, His204, Cys207, Glu217 and Asp227, dampened zinc(II)-ion sensitivity or completely ablated zinc(II)-stimulation capacity (Tab. 1).

### Cell Surface Expression of Mutated mGPR83

The majority of the mGPR83 variants exhibited a 70% or more cell surface expression compared with wild type mGPR83 (Tab. 1). However, three of the eleven mutants (Glu217Ala, His204Ala and His321Ala) were only moderately expressed, with expression levels between 30-50% as compared to wild type mGPR83. In comparison, the constitutively active Cys304Trp mutant in TMH6 was characterized by an expression level of 87.9 ± 6.0% in comparison to 100% wild type (Tab. 1).

### Constitutive Activation of mGPR83 by Mutations

Surprisingly, the alanine mutagenesis at potential zinc(II) binding sites ultimately led to the identification of several constitutively active mutants, in particular His27Ala, Asp218Ala, Glu230Ala and His321Ala. All of these mutations increased the basal signaling level (IP_3_ formation) by 133-150% compared to wild type (Tab. 1). Mapping of these amino acids to the structural mGPR83 homology model revealed a spatial clustering of these amino acids in a region between ECL2/ECL3 and TMH6 (Fig. 3). This spatial receptor region is well characterized for other GPCRs to be sensitive for signaling activity, especially in their interplay [49]-[53]. However, this spatial region’s regulatory role for mGPR83 activity is determined in detail for the first time here. Specifically, substitution of His27Ala increased basal activity by 133% compared to wild type (Tab. 1). This residue is located at the extreme N-terminus in close proximity to the signal peptide (positions 1-16) and is not included in the structural homology model because a structural fragment template is lacking. This is the first indication that the N-terminus of mGPR83 participates in signaling regulation.

### Homodimerization of mGPR83

**Figure 4:**
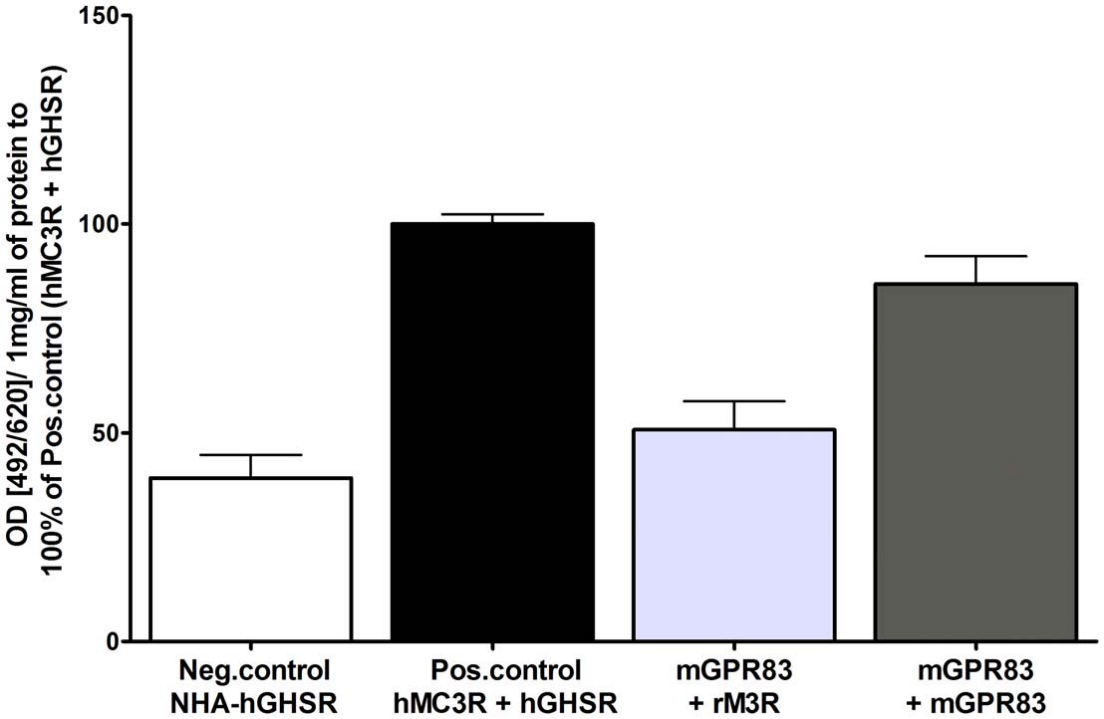
Homodimerization of mGPR83. Dimerization studies were performed using sandwich ELISA. COS-7 cells were transiently transfected. As negative control serves the NHA-hGHSR (white column) and as positive control the co-transfection of NHA-tagged hMC3R and FLAG-tagged hGHSR (black column, [28]). The light grey column represents the average of the HA- respectively FLAG-tagged mGPR83 in combination with the correspondent tagged rM3R. The dark grey column represents co-transfection of HA-tagged mGPR83 and FLAG-tagged mGPR83. Dimerization was measured via the HA epitope. The mean absorption (492 nm/620 nm) is calculated per 1 mg/ml of protein and shown as percentage of the hMC3R/hGHSR heterodimer (absorption (492/620)/ 1mg/ml of protein: 0.3 ± 0.04). Data were assessed from 3 independent experiments, each performed in triplicates and represent mean ± SEM.

Finally, we assessed the capacity of mGPR83 to form homodimeric complexes, which is an important and common feature of GPCRs [54], [55]. To analyze the capacity of mGPR83 to homodimerize, a sandwich ELISA was performed. A homodimeric association of mGPR83 (dark grey column, Fig. 4) was observed, which is comparable to the control heterodimer of hMC3R/hGHSR (black column, Fig. 4). For verification, we analyzed the interaction with the rat M3R (light grey column, Fig. 4). This signal is comparable to the negative control (white column, Fig. 4), thus corroborating significant mGPR83 dimer formation.

## Discussion

The major aim of this study was to gain novel insights into the molecular underpinnings of mGPR83 signaling pathway(s) without existing knowledge of an endogenous ligand. Our results demonstrate that mGPR83 is characterized by a basal level of Gq/11 mediated accumulation of inositol trisphosphate. This information is of particular importance for future studies regarding the directed potential in pharmacological interventions targeting this receptor. Of important note, our finding that mGPR83 is an Gq/11 signaling GPCR was also confirmed by identification of several CAMs and the capacity to be activated by zinc(II)-ions.

### GPR83 Activates the Gq/11 Mediated Pathway by Zinc(II)-ion Binding at Specific Signaling Sensitive Amino Acids

Zinc(II) is one of the most abundant trace elements in the human body, plays a prominent role in human health and interact with many GPCRs. Zinc(II)-ions are stored in glutamatergic synaptic vesicles and are co-released with neurotransmitters into the synaptic cleft where zinc(II) concentrations up to 300 µM can be obtained [56], [57]. Within the neuronal expressed GPCRs it could be shown that zinc(II) activates and/or potentiates MC4R, β2-adrenergic receptor and tachykinin NK3 receptor signaling, also by engineered metal ion sites [20], [23], [25], [26]. In contrast, the µ-opioid receptor and the D_2_ or D_4_ dopamine receptors are inhibited by zinc(II)-ions [47], [58]. Zinc(II)-ions can act as signaling molecules by catalytic and structural effects and it is known that different cell-surface proteins, notably in neurons are effected by metal-ions like transporters for neurotransmitters, ion channels or even GPCRs [59], [60].

Strikingly, we here show for the first time that zinc(II) acts agonistically in a micro-molar range on mGPR83. Already concentrations of 1nM Zn(II) lead to a first slight increase in mGPR83-signaling which is maintained until concentrations up to 10 µM zinc(II). From this first activation-level a strong and steeply stimulation of mGPR83 can be reached (Fig.2) in accordance to the obtained physiological concentrations in the synaptic cleft at around 100 µM Zn(II). Extracellular Zn(II)-concentrations above 1 mM lead in cell culture to cell-death. In multicellular organisms virtually all zinc is intracellularly located and appears in complexes with proteins and nucleic acids [61]. In accordance with the fact that physiologically only 1% of all Zn(II) occurs extracellular, to high concentrations of administered Zn(II) lead to a modified pH value of the medium and therefore to cell death *in vitro*.

By site-directed mutagenesis, the zinc(II)-sensitive amino acids in the extracellular domain, which were predicted according to known binding motifs and implications from a structural homology model, were explored. Typical zinc(II)-ion binding motifs are constituted by combinations of Cys-Cys-Cys-Cys, Cys-His-Cys, His-His-His or His-Glu-His side-chains [48]. Our studies elucidated a few specific amino acids (His42, His145, His204, Cys207, Glu217, Asp227) participating in zinc(II)-binding. Alanine mutations at these positions led to a loss of zinc(II)-mediated activity. Furthermore, the identification of six zinc(II)-sensitive positions opens the possibility that zinc(II) binds at two different clusters within the spatial region between the Ntt, ECL2, and TMH3/4. This finding is in accordance to a biphasic stimulation curve of zinc(II) at mGPR83 (Fig. 2).

In accordance to our structural homology model, side-chains of residues Glu217 and Asp227 are keeping the ECL2 in its conformation, presumably through hydrogen bonds within the backbone of this loop (Fig. 3). Binding of zinc(II) between those residues likely causes an interruption of stabilizing intramolecular interactions in the inactive state. Consequently, zinc(II)-binding induces structural shifts between the ECL2 and may alter its interaction with other domains. This potentially can modify the precise juxtaposition of ECL2, which has been identified as a crucial activation step for other family A GPCRs [49]-[53], [62], [63]. A potential relationship between homodimeric mGPR83 variants and zinc(II)-binding properties have yet to be elucidated because details of the homodimer interface are uncharacterized. However, the aspect of zinc(II)-induced mGPR83 activation might be of high future interests. This includes the physiological impact of GPR83 in relation to zinc(II)-ion availability, but also a potential modulatory function of zinc(II) on other ligand-receptor interactions. Zinc(II) could be of catalytic or structural impact for an undiscovered physiological mGPR83-ligand. Additionally, potential interactions of mGPR83 with other proteins (for example GPCRs) could be influenced structural and/or functional by zinc(II).

In addition to our findings of Zn(II)-stimulation on mGPR83 we have shown that calcium(II) and magnesium(II) did not induce Gq/11-signaling when administered in physiological concentrations (as occurring in the synaptic cleft) [64]-[66]. This finding might be related to missing binding motifs for other divalent ions than zinc and support an exclusive stimulation of GPR83 by zinc.”

### First Identification of Structural Components Important for Signaling Regulation

Several mutated mGPR83 variants (e.g. Asp218Ala, Glu230Ala, His321Ala) exhibited varying degrees of ligand-independent basal activity. The mutated residues are distributed in a cluster-like manner in a spatial region between ECLs 2/3 and TMH7 (Fig. 3). Of an important note, the level of constitutive signaling did not allow further stimulation by zinc(II) (Tab. 1). This implies that the CAM positions identified here could participate in zinc(II)-binding and receptor activation and that loss-of-function mutations cannot be induced because of the overriding constitutive activity. However, these CAMs indicate a region of sensitivity for receptor activation that is located in the extracellular domain and TMH7, which is close to the identified zinc(II)-binding sites.

Interestingly, the occurrence of a CAM (His27Ala) and a zinc(II)-binding sensitive residue (His42Ala) at the extreme extracellular N-terminus of mGPR83 highlights this region as a determinant for signaling regulation. This region of mGPR83 is composed of nearly 70 amino acids that are conserved among species (Electronic supplementary material, Fig. S1). Furthermore, these structural findings support a potential domain-like fold in this region. It is of future interest to take these first hints into consideration for studying signaling mechanisms of the GPR83 in detail. This N-terminal part might function as a tethered inverse agonist that switches to an intramolecular agonist during activation or may function as a signal transmitter. Such scenario for a family A GPCR has been investigated at the extracellular part of glycoprotein hormone receptors (reviewed in [67]).

### The mGPR83 Forms Higher Order Complexes

Knowledge concerning the determinants important for GPR83 activation, modulatory or agonistic effects of metal-ions, and mechanisms of signal transformation from an extracellular stimulus to initiation of an intracellular cascade is a prerequisite to estimate its full functional and pharmacological capacity. This includes the identification of protein-protein interactions for example with other GPCRs. Our data indicate mGPR83 forms homodimeric, or higher order, complexes but the exact identity of these complexes have yet to be characterized. It is well reported that oligomeric associations could have dramatic influences on signaling properties or pharmacological features of GPCRs [54], [55]. For instance, the orientation of transmembrane helix 4 is modified during activation of dopamine receptor homodimers [68]. Additionally, activation of a single constituent of a heterodimeric complex of α2-adrenergic and µ-opioid receptors can trans-incativate the second receptor [69]. Activation of the dimeric metabotropic glutamate receptor is related to helical intersubunit re-arrangements [70]. In many scenarios of dimeric GPCR-GPCR interrelation the ligand binding capacity is especially influenced [55]. Reflecting the enormous potential impact of dimerization on structure and function of GPCRs, this characteristic might also be considered a prominent mechanistic feature for GPR83. Future studies should tackle specifically this issue more detailed.

In conclusion, this study revealed that mGPR83 exhibits a basal level of Gq/11 meditated IP_3_ signaling, which can be increased by mutations at certain positions in the transmembrane region. Furthermore, we show that mGPR83 can be stimulated by zinc(II)-ions, and we identified detailed binding site residues within the N-terminal domain, which suggests a regulatory role for signal transformation. In addition, mGPR83 is able to form homodimeric or oligomeric complexes, which can have profound pharmacological prospective. This feature also suggests that GPR83 has an interplay capacity with other GPCRs. Ultimately, we believe this study opens the field for further studies to unravel the physiological role of GPR83. Additionally, the study design described herein, which is the first to investigate ligand-independent signaling mechanisms, may serve as a roadmap to characterize other orphan GPCRs.

## Supporting Information

Figure S1 **Sequence alignment comparison between particular family A GPCRs.** The amino acid sequences of GPR83 from different species are represented in comparison with bovine rhodopsin and the human beta-2 adrenergic receptor. The crystal structure of rhodopsin was used as a template for structural GPR83 homology model. Based on the crystal structures of rhodopsin and the beta-2 adrenergic receptor the structural dimensions of the helices and loops are assigned (lilac boxes). Similar residues regarding biophysical properties are marked with gray background (blossum 62 matrix). Color code: black – proline, blue – positively charged, cyan/green – aromatic and hydrophobic, green – hydrophobic, red – negatively charged, gray – hydrophilic, dark-red – cysteines, magenta – histidine. The extracellular CAM H27A was identified in this project.
(TIF)

Figure S2 **mGPR83 shows no basal activity in Gs or Gi.** COS-7 cells were transiently transfected with the empty expression vector pcDps (mock), pcDps carrying the wild type mGpr83, the mGpr83 C304W mutant, the hTSHR or the h5HTR1B. Two days after transfection, stimulation with 50 µM forskolin, 100 mU/ml bTSH and 100 nM serotonin was carried out, cells were lysed and cAMP-accumulation was measured. The bTSH stimulated hTSHR serves as Gs positive control (dark grey column, [23], [24]), the forskolin serotonin co-stimulated h5HTR1B as Gi positive control (light grey column, [25]). Data were evaluated from 3 independent experiments, each performed at least in triplicates and calculated fold over the (basal) mock transfection with 2.6 ± 0.1 nM cAMP set to 1. Shown data represent mean ± SEM.
(TIF)

Figure S3 **Concentration-response curve of calcium(II)- and magnesium(II)- stimulation at mGPR83.** HEK293 cells were transiently transfected with the empty expression vector pcDps (mock) or pcDps encoding the wild type *Gpr83*. Medium without additives contains 1.8 mM Ca(II) and 0.4 mM Mg(II) (matches the basal values). Two days after transfection, stimulation with calcium(II) up to 4.8 mM **(A)** and magnesium(II) up to 8 mM **(B)** was carried out, cells were lysed and IP_3_-accumulation was measured in a reporter gene assay. The hTSHR stimulated with 100 mU/ml bTSH functioned as assay control (data not shown, [31], [32]). Data were evaluated from 3 independent experiments, each performed at least in triplicates and calculated fold over the mock transfection, with 10496.7 ± 484.2 for Ca(II) and 16206.7 ± 1784.8 for Mg(II) relative light units, set to 1. Shown data represent mean ± SEM.
(TIF)
