# Supplementary figures and images for: G-Protein Coupled Receptor 83 (GPR83) Signaling Determined by Constitutive and Zinc(II)-Induced Activity
Source: PLoS One. 2013 Jan 15;8(1):e53347. doi: 10.1371/journal.pone.0053347 (PMC3546042; doi:10.1371/journal.pone.0053347)

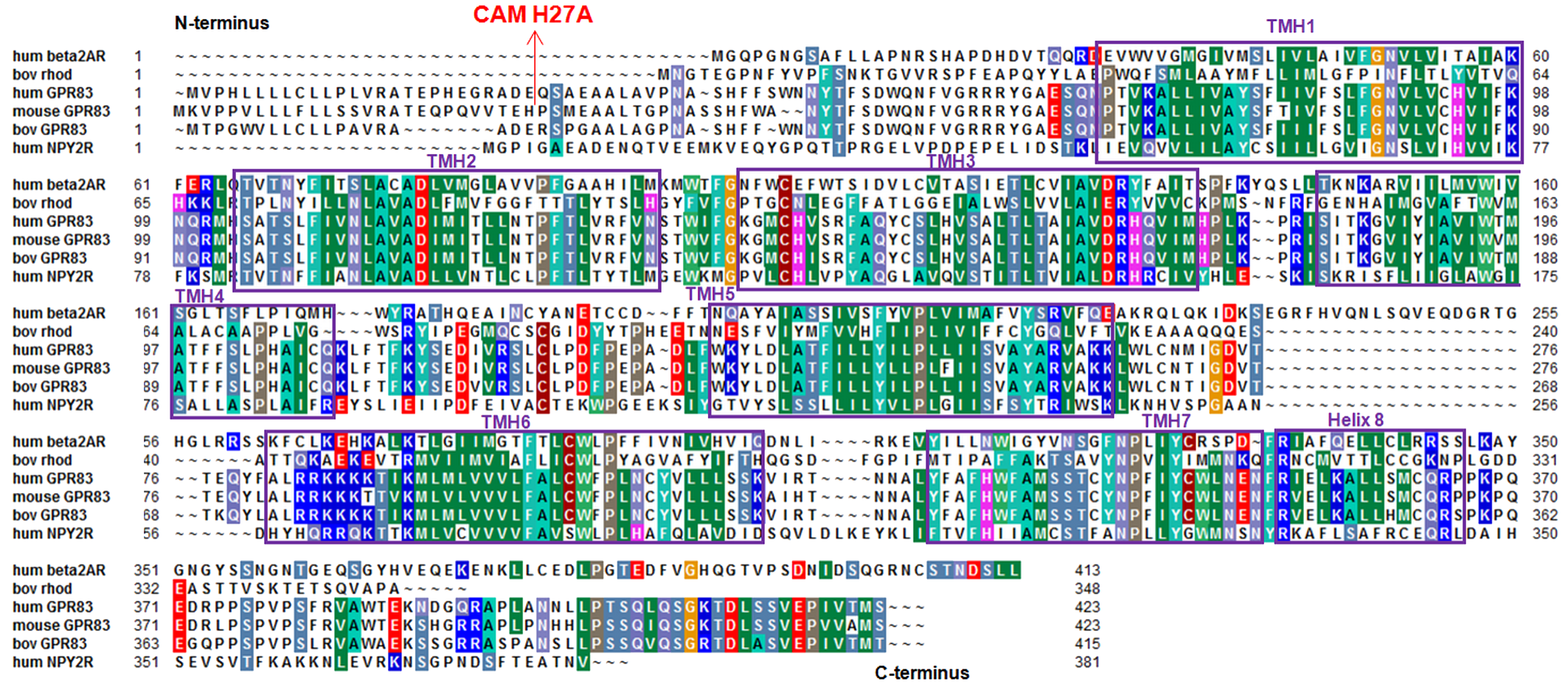

Supplement: Figure S1 — Sequence alignment comparison between particular family A GPCRs. The amino acid sequences of GPR83 from different species are represented in comparison with bovine rhodopsin and the human beta-2 adrenergic receptor. The crystal structure of rhodopsin was used as a template for structural GPR83 homology model. Based on the crystal structures of rhodopsin and the beta-2 adrenergic receptor the structural dimensions of the helices and loops are assigned (lilac boxes). Similar residues regarding biophysical properties are marked with gray background (blossum 62 matrix). Color code: black – proline, blue – positively charged, cyan/green – aromatic and hydrophobic, green – hydrophobic, red – negatively charged, gray – hydrophilic, dark-red – cysteines, magenta – histidine. The extracellular CAM H27A was identified in this project. (TIF) [file pone.0053347.s001.tif]

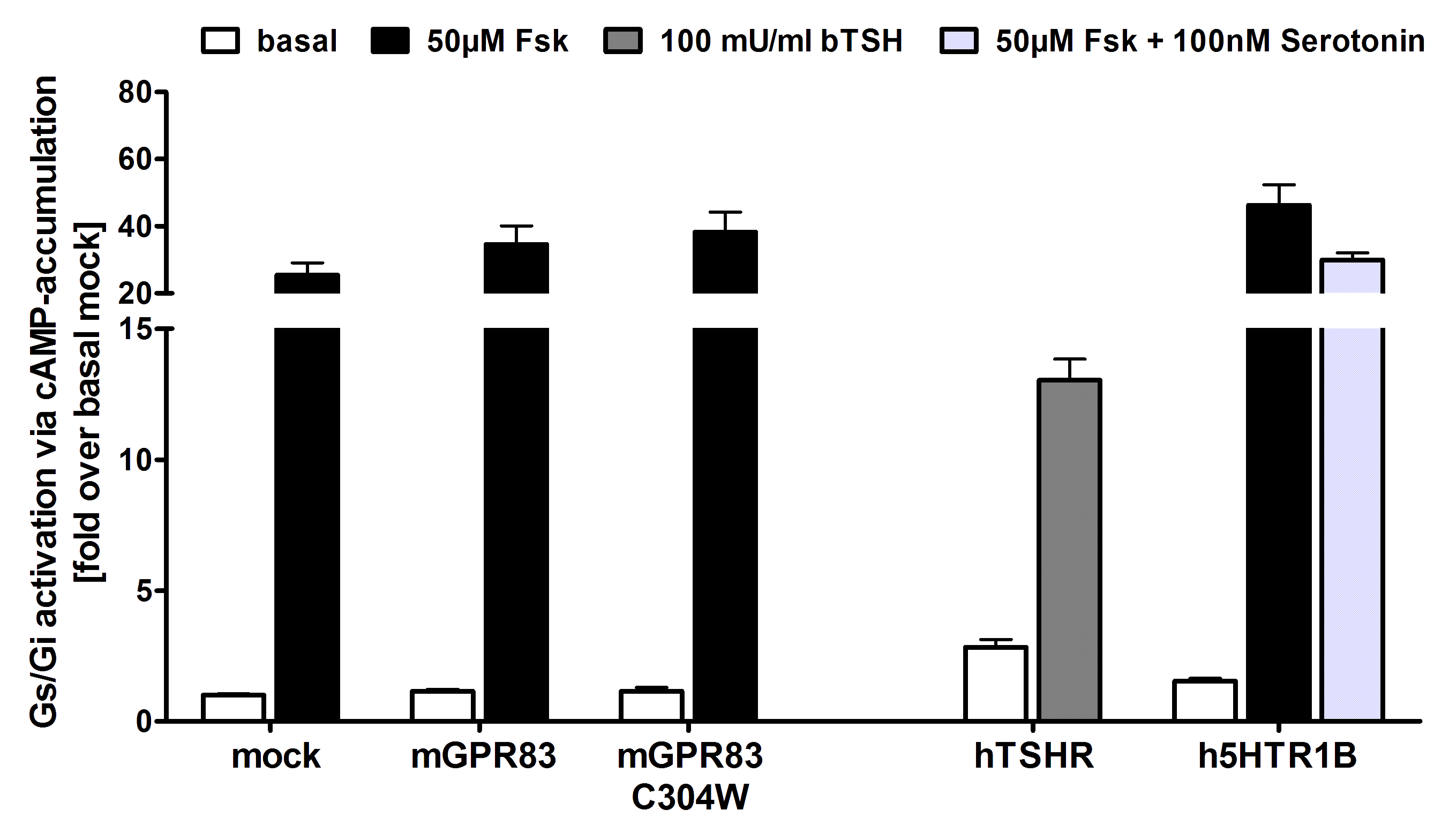

Supplement: Figure S2 — mGPR83 shows no basal activity in Gs or Gi. COS-7 cells were transiently transfected with the empty expression vector pcDps (mock), pcDps carrying the wild type mGpr83, the mGpr83 C304W mutant, the hTSHR or the h5HTR1B. Two days after transfection, stimulation with 50 µM forskolin, 100 mU/ml bTSH and 100 nM serotonin was carried out, cells were lysed and cAMP-accumulation was measured. The bTSH stimulated hTSHR serves as Gs positive control (dark grey column, [23], [24]), the forskolin serotonin co-stimulated h5HTR1B as Gi positive control (light grey column, [25]). Data were evaluated from 3 independent experiments, each performed at least in triplicates and calculated fold over the (basal) mock transfection with 2.6 ± 0.1 nM cAMP set to 1. Shown data represent mean ± SEM. (TIF) [file pone.0053347.s002.tif]

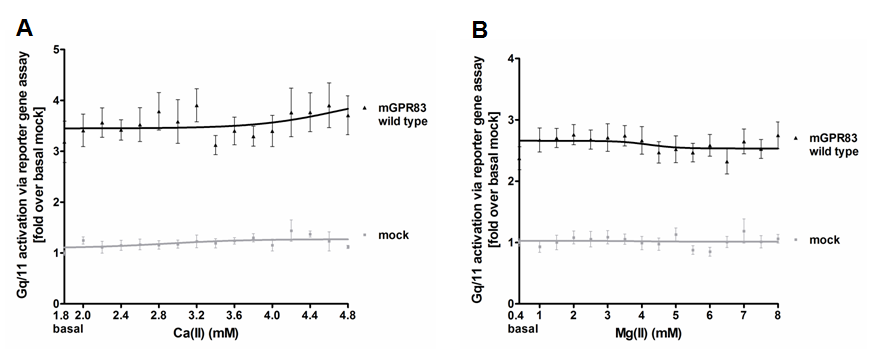

Supplement: Figure S3 — Concentration-response curve of calcium(II)- and magnesium(II)- stimulation at mGPR83. HEK293 cells were transiently transfected with the empty expression vector pcDps (mock) or pcDps encoding the wild type Gpr83. Medium without additives contains 1.8 mM Ca(II) and 0.4 mM Mg(II) (matches the basal values). Two days after transfection, stimulation with calcium(II) up to 4.8 mM (A) and magnesium(II) up to 8 mM (B) was carried out, cells were lysed and IP3-accumulation was measured in a reporter gene assay. The hTSHR stimulated with 100 mU/ml bTSH functioned as assay control (data not shown, [31], [32]). Data were evaluated from 3 independent experiments, each performed at least in triplicates and calculated fold over the mock transfection, with 10496.7 ± 484.2 for Ca(II) and 16206.7 ± 1784.8 for Mg(II) relative light units, set to 1. Shown data represent mean ± SEM. (TIF) [file pone.0053347.s003.tif]
